# Discovering the Value Creation System in IoT Ecosystems

**DOI:** 10.3390/s21020328

**Published:** 2021-01-06

**Authors:** Carlos Alberto Lopez, Luis Fernando Castillo, Juan M. Corchado

**Affiliations:** 1 Industrial Engineering Department, Facultad de Ingeniería y Arquitectura, Universidad Nacional de Colombia, Manizales 170003, Caldas, Colombia; caralopezcas@unal.edu.co; 2 Grupo de Investigación Inteligencia Artificial, Departamento de Sistemas e Informática, Facultad de Ingenierías, Universidad de Caldas, Manizales 170004, Caldas, Colombia; 3 Departamento de Ciencias Computacionales, Doctorado en Ciencias Cognitivas, Universidad Autónoma de Manizales, Manizales 170001, Caldas, Colombia; 4 Air Institute, IoT Digital Innovation Hub, 37188 Salamanca, Spain; corchado@usal.es; 5 Bisite Research Group, University of Salamanca, 37007 Salamanca, Spain; 6 Department of Electronics, Information and Communication, Faculty of Engineering, Osaka Institute ofTechnology, Osaka 535-8585, Japan; 7 Pusat Komputeran dan Informatik, Universiti Malaysia Kelantan, Kelantan 16100, Malaysia

**Keywords:** value creation, IoT ecosystem, value offer, system dynamics, value capture, business model

## Abstract

Internet of Things (IoT) should not be seen only as a cost reduction mechanism for manufacturing companies; instead, it should be seen as the basis for transition to a new business model that monetizes the data from an intelligent ecosystem. In this regard, deciphering the operation of the value creation system and finding the balance between the digital strategy and the deployment of technological platforms, are the main motivations behind this research. To achieve the proposed objectives, systems theory has been adopted in the conceptualization stage, later, fuzzy logic has been used to structure a subsystem for the evaluation of input parameters. Subsequently, system dynamics have been used to build a computational representation and later, through dynamic simulation, the model has been adjusted according to iterations and the identified limits of the system. Finally, with the obtained set of results, different value creation and capture behaviors have been identified. The simulation model, based on the conceptualization of the system and the mathematical representation of the value function, allows to establish a frame of reference for the evaluation of the behaviour of IoT ecosystems in the context of the connected home.

## 1. Introduction

In the context of a Connected Home, the Internet of Things (IoT) ecosystem could be defined as a special type of business ecosystem, which is composed of a group of enterprises and individuals that compete and cooperate in a socioeconomic environment, using a common group of central assets. These assets are associated with the communication between the physical world of things and the virtual world of the Internet [1]; Regarding this concept of communication, it has evolved into a framework that takes advantage of the availability of heterogeneous gadgets (sensors) and interconnection solutions, as well as augmented physical objects (home appliances) that provide an information base shared worldwide. Its ultimate goal is to support the application design that involves both people and object representations at the same virtual level [2].

For their part, Tafti, Kordnaeij [3] argue that in an ecosystem, not only competence but also cooperation increases efficiency and improves business performance; considering this statement and the literature review, it is evident that IoT ecosystems in Connected Homes are at a “birth” stage, where, on a scientific and practical level the bases of a value proposition for new products and services are laid; as well as the best way to deliver them to customers [4].

In line with the above, the main implication for white goods companies goes through the unification of two ecosystems that have been separated over decades; according to Iansiti and Lakhani [5] production and consumption ecosystems merge into one, in other words, a manufacturing enterprise value chain enters into the consumers’ homes (it becomes pervasive), through the fabrication of “smart” and connected appliances (augmented), which open a doorway to the design of new models of digital business; so that these enterprises’ value offer further evolves the simple selling and post selling service of consumer durable goods [6].

Nonetheless, there are several concerns regarding the use and destiny of the data resulting from ecosystem interactions; in fact, it is a principal adoption barrier for the technological advances in this area. Assuming that future solutions will support the appropriation of those advances [7,8], the white goods companies understanding of the users’ concern regarding data will leverage the social and sustainable development of IoT ecosystems [9].

Thus, the transformation of the white goods industry is based on the development of digitalization capacities in the production ecosystem, as well as the new business model explorations in the consumption ecosystem [10]. Two key elements are an integral part of this digitalization; the first one is related to the organizational architecture, by which the enterprises aim to break the traditional silo architecture, to achieve agile and innovative organizations, adapted to the new environment. This also leads these companies to redefine their ambitions and develop new digital operating models [11].

**Figure 1:**
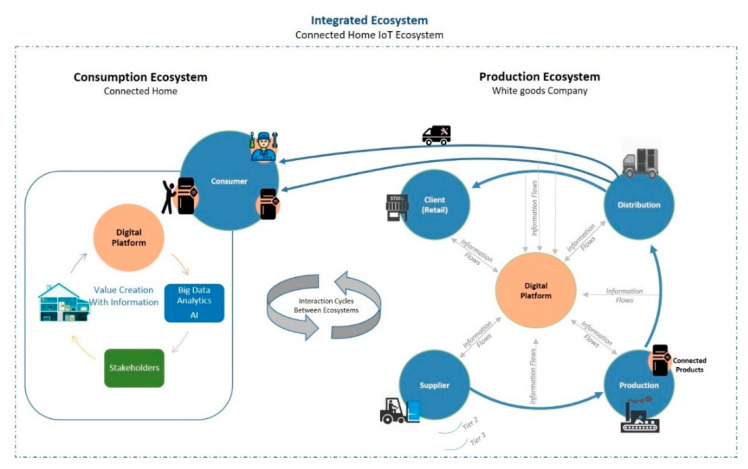
Integrated Ecosystem.

The second element is composed by the technological enablers, which the investigators view as a lever for business model innovation [12]; In this regard, several technological tendencies stand out; although, the common denominator in the different investigations could be summarized in two, the technologies related to advanced data analytics [13], and the artificial intelligence applied along the supply chain [14,15]. The new interrelations propelled by the digital technologies in the white goods industry could be analysed more optimally by differentiating between the production ecosystem and the consumption ecosystem. The production ecosystem consists in the interrelations that happen in a value chain, as the production and selling of a product or the post selling service. On the contrary, the consumption ecosystems consist in the interrelations that evolve after selling a product or offering a service digitally [16,17]. For better understanding, Figure 1 illustrates the integration of these two concepts.

To develop an architecture for the design of the IoT ecosystems of Connected Homes, its methodological and technological aspects must be defined. To this end, it was necessary to build mathematical models that would include the descriptive variables of the ecosystem. Some authors have proposed approaches which used the could computing paradigm, as well as its extension, called “Fog Computing”, where data processing and applications are concentrated in the devices at the edge of the network, and not in the cloud [18].

In line with the architecture, Abdmeziem, Tandjaoui [19] propose a classification of three categories for the different technologies that are part of IoT: First, the sensing technologies, with which the data is collected, then the middleware layer, which is in charge of processing and managing the data, and lastly the performance technologies, which represent the physical extension of IoT applications. These categories allow us to infer the need for a data analysis architecture in the design of the platform, through the application of advanced analytics.

In short, architectures are an important means of selecting the correct combination of technologies, which results in the creation and capture of value in the ecosystem [20]. Therefore, value is created when an architecture is scalable, when it adapts to the needs of the interest groups, and when they are resilient thanks to good management. In this regard, an optimal architecture also reduces the Time to Market of the platform and its associated computer applications [21].

## 2. IoT Home Market Evolution

**Figure 2:**
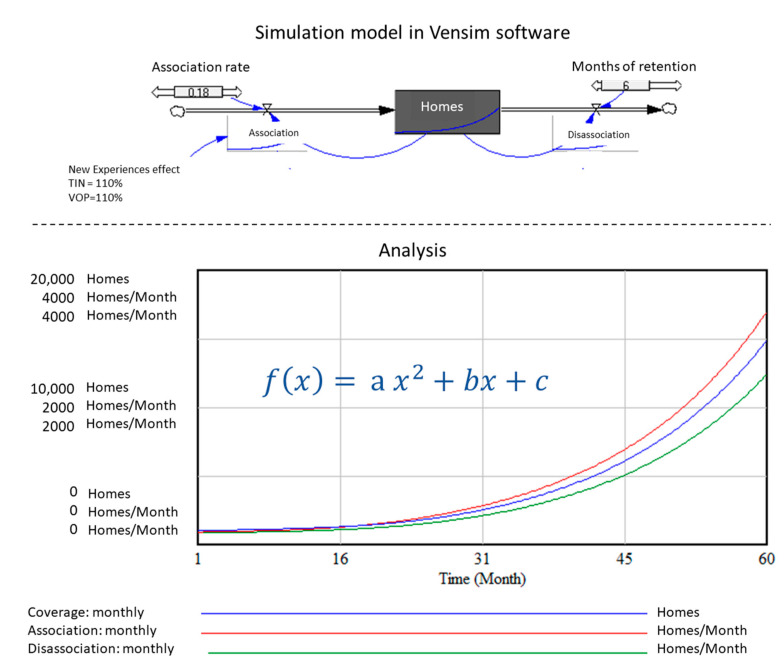
Forecast of the number of connected Homes.

Inside the IoT ecosystem, the connection of homes to the new digital services grows dynamically over time. According to APPLIA (European association of home appliance manufacturers) the number of connected homes in Europe will grow 4X by 2024, compared to the 18 million reported in 2017 [22]. In this regard, there are different ways to calculate this connection and verify the shape of the curve that represents this growth. According to a simple dynamic simulation model (up to 60 months), which relates the number of connected homes by means of an association and disassociation rate; as shown in Figure 2, the curves of the new homes in the network and the disassociated homes are in constant growth. Thus, it is possible to infer that the number of users that are associated, will continue to be significantly bigger that of those who are disassociated. The network effect is created through ecosystem adoption, leading to value creation.

Analyzing the curve, it can be observed that the number of connections increases over time, taking the shape of a quadratic function ($x^{2})$. Regarding the approximation of network growth, it does not give detailed explanation as to network behaviour, especially because of the supposition that the networks of connected homes are influenced by externalities. According to Larrosa [23], these externalities are caused by the limitations of valuing laws; so that it is inferred that laws such as Metcalfe’s require several assumptions that limit the projections. Nonetheless, this is an initial analysis and it could be argued that the connected homes’ growth curve is of interest to white goods companies. From a theoretical point of view, the development of home-related solutions has a lot of potential, this topic will be addressed in-depth in the network effect section.

Similarly, the Perceived Value concept that has been widely studied by marketing and business investigators, is referred to as a general evaluation made by the users of a service or product [24]; which can be summarized as the discrepancy between the benefits that are obtained and the costs of those benefits. In the existing literature, different approaches have been presented for different analysis areas, whose goal is to both define the meaning of the concept and to find a way to increase its value through an effective structured value offer [25].

In this regard, it has been possible to confirm the existence of different work frames that allow for the generation of value offers [26], Alternatively, in this investigation an expert panel was developed, where two values emerged that are predominant in terms of system evolution (social and economic value) [27]. According to the literature, these values are classified as extrinsic perceived values if the objective orientation perspective is considered [28]. Thus, the definition of value creation in the IoT ecosystem agreed by the experts at an innovation workshop between the years 2018 and 2019, is the following: “Economic and social value creation through the design of a digital value proposal for the consumer, which is based on the data exploitation and the business models adaptation and, thus, it helps to improve the resiliency and the growth of the IoT ecosystem in Connected Homes.”

In this paper our hypothesis states that the business models represent a new analysis unit, and thus illustrate a new holistic vision on the enterprises’ business approach. Nevertheless, white goods companies face the same issues as start-ups, they must define and develop a digital value proposal, which is essential in the launching phase and allows start-ups to connect their transformation ideas in the market [29].

Similarly, innovation leveraged by new technologies is an essential element of any competitive initiative, especially in today’s markets, which by nature are uncertain and where the brand value, the quality or the manufacturing capabilities are not enough to attract new consumers. Van Le and Suh [30] investigations confirmed that value proportions have evolved in the last decade and have focused on keeping in the center the client or consumer’s perspective at the core.

White goods companies have started digital transformation processes, leading them to create new value offers, which to a large extent have focused on operational efficiency [31]. This praxis proves that these companies have shied away from offering digital solutions directly to the final consumer. Hence, understanding the value creation system of the IoT ecosystem is a big step forward in the innovation of business models and their value offer. In the context of this investigation, it is necessary to highlight that an IoT ecosystem is defined as a business ecosystem that may affect the value creation system either positively or negatively.

**Figure 3:**
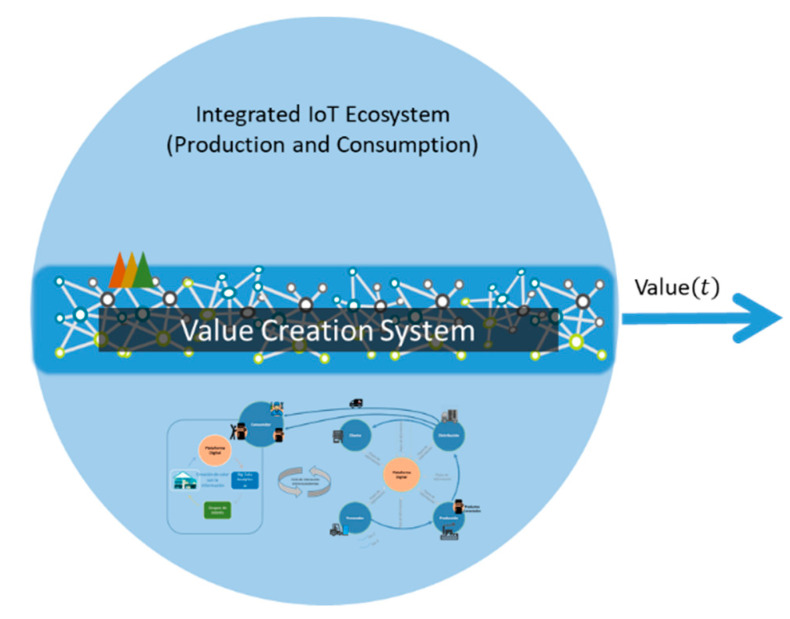
Vision of value creation System.

From the perspective of system theory, inside the IoT ecosystem there may be a special system that is in charge of producing the value (See Figure 3), which dynamically benefits from the connection of a large amount of gadgets that conform the corporate ecosystem of a white goods company [32], to connect the unconnected allows the information of a whole supply chain to be collected and an analysis process to be nurtured [33,34,35]; the ultimate goal of creating “Actionable intelligence”. Thus, it can be inferred that the value creation system is the engine of the whole ecosystem, which generates actionable intelligence to make the business and the operative models work synchronously. Similarly, the engine is defined as the configuration of these elements.

## 3. IoT Home Analysis

To analyze the behaviour of the system for the creation of value in the IoT ecosystem, the use of simulation techniques has been defined in the methodological design. This makes it possible to assess the behaviour of the system (which has been done by experts) in terms of value creation. In this line, despite the computational modelling challenges that complex systems represent, dynamic simulation is an ideal tool for building models that reproduce the behaviour of complex systems [36,37,38]; However, there is no universally accepted evaluation method for validating a dynamic model. Thus, the approach used in this research is to check the results in practice, using expert opinion.

It has not been possible to find any reference models on which to base the representation of the value creation system of the IoT ecosystem. Likewise, it has not been possible to compare it with a model that would be structured as a real system. With this in mind, to build the model, an exhaustive document analysis has been carried out, which together with a panel of first-level experts, was used to conceptualize the model, as well as the mechanisms for performing simulations and verifying the results.

Therefore, once the model of the value creation system was built, it was simulated using a set of alternative assumptions, giving rise to a set of results in each simulation. Then, a comparative analysis of the different scenarios has been conducted, enabling the authors to choose the most promising one, in accordance with the objectives pursued by white goods companies

**Figure 4:**
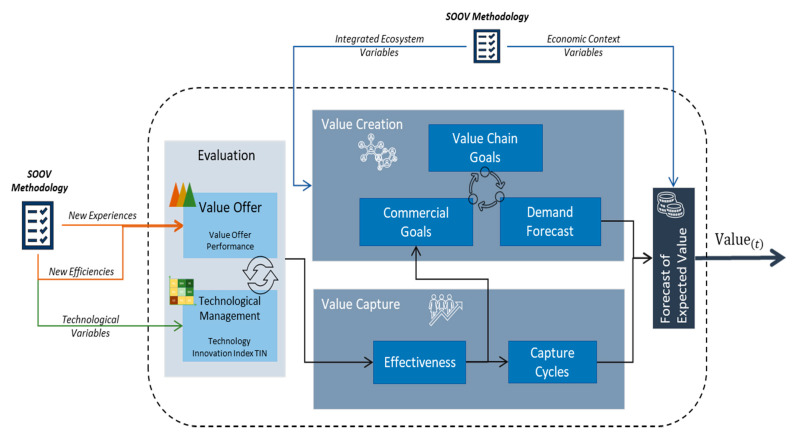
Conceptual diagram of value creation system.

Having reviewed the background literature, the elements that make up the value creation system have been identified and are given in Figure 4.

The value creation system is composed by four elements, which are seen as subsystems or states through which the information passes, these states start with a valued supply evaluation process from two fronts, the value offer in itself, as well as the evaluation of the technological management that supports that offer. It can be observed in the image, in the value capture state, that the combination of the entrance elements affects the performance of the system positively and negatively. In this part, a model that allows to evaluate the behaviour of the capture has been designed, which could be expressed as the capacity of the value capture offer.

The next state is value creation, which is related to demand, as discovered in the background literature review. Hence, the system is modelled through the interaction of three key elements: The commercial objectives of the value offer, the supply chain’s objectives to deliver the product or service and the calculus of the demand forecast. In this regard, it is possible to revise the behaviour of the network effect for a specific value offer. It is necessary to highlight that, some context variables of the ecosystem, such as the ones related to the number of people per family, the number of business partners in a company and the market share, are used to adjust the behaviour of the network effect of the system to the existing variables in the real world.

Finally, the last subsystem is related to the calculus of the total value that is created in the system and the quantity captured by the entrance scenario. The combination of the creation process with the capture process will lead to the creation of some forecasts based on the interaction dynamics of the subsystems.

### 3.1. Evaluation Subsystem

The concept of the evaluation subsystem was conceived as a two-part review process that combines three input elements, the first part deals with the evaluation of the Technological Management for a digital initiative, to do this a composite index was built; the second is defined as the evaluation of the performance of the digital value offer through the new efficiencies and new experiences generated, for this a fuzzy logic model was developed. The outputs of this subsystem allow to simulate the behaviour of the creation of value through a dynamic model presented later in this paper.

#### 3.1.1. Technology Innovation Index

Saunders and Brynjolfsson [39] investigations confirmed that technology boosts value creation up to 52% over the average in traditional companies (including white goods manufacturers). Abood and Quilligan [40] argue that the correct technology combination in the companies generates new experiences and new efficiencies that help re-invent the operative model.

Considering that white goods companies are facing a changing and competitive environment, innovation in information technology seems to be the best way to deal with the big challenges in this type of environment. Thus, correct technology management will allow these companies to adapt their way of work [41,42].

To try to measure this impact on the value creation system, an index that combines three variables has been developed: the “correct combination of technologies” in the platform; the “resiliency”, understood as the capacity of the platform to quickly retake normal operations after a contingency—this variable arose from an expert study and became one of the most relevant ones in 2020 due to the impact of the pandemic on the industry and organizations- [13,43], and lastly, the level of “investment” that is projected over the platform [40].

If correct technology management increases and lowers the value, the description in this index can be distributed in the following percentage value range:

Technology Innovation Index = TIN = value between (80% and 120%).

#### 3.1.2. Value Offer Performance

Considering the uncertainty of the value creation system, where there is no complete factor list to consider for the problem domain and, since there is no known theoretical model to mold it, it was necessary to apply fuzzy logic in this investigation; as Zadeh [44] mentioned: “As the complexity increases, the precise declarations lose their meaning and the meaningful declarations lose precision”, so to mold the value offer performance (VOP) in the value creation system in the IoT ecosystem, an evaluation model based on fuzzy logic has been built [45,46].

This model solves the problem associated with identifying the perfect performance of the Value Offer; given a group of entry variables (entry space), the exit variables have the correct value (exit space). Thus, fuzzy logic is the appropriate way to map these variables. That said, according to Abood and Quilligan [40] the effectiveness of the value offered in the digital business models is forged thanks to the new experiences and the new efficiencies created for the client and consumer, with this in mind, they have defined the following elements in the fuzzy logic language.

#### 3.1.3. Discourse Universe and Linguistic Variable

Value Offer Performance concept was defined as linguistic variable, which will be forecast based on fuzzy logic whilst, the diffusion universe. Thus, Table 1 gives the value range expressed by the property processes through the linguistic variable. It cannot be forgotten that a value for example of 85% has a low value percentage and an acceptable one, which ultimately is the essence of fuzzy sets, the different levels of membership, which depend on the established rules.

#### 3.1.4. Membership Function

The Fuzzification process consists in taking the entries sharp values and determining the degree of belonging to the new associated fuzzy groups. The degree is determined through the belonging function for the new experiences and the efficiencies of the group, which indicates the degree of belonging to a group of each element in a given universe.

**Figure 5:**
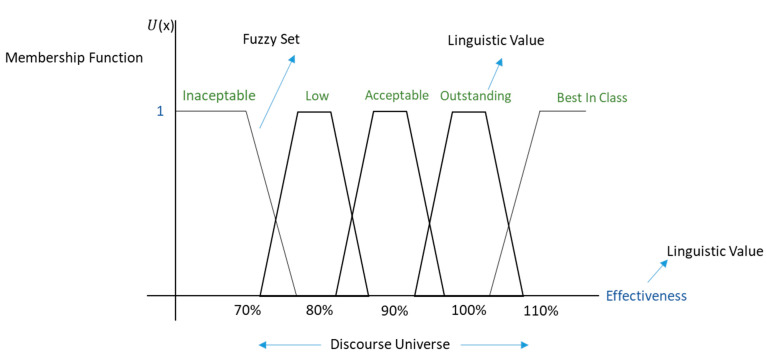
Value offer performance (VOP) Fuzzy classification.

In line with the above, the change of membership function from one set to another is gradual and limited by the lack of well-defined boundaries between these sets. The following membership function has been defined to indicate the degree to which each element of the universe of discourse (Effectiveness = EF), belongs to a set. In other words, the membership function of a set FG (Fuzzy Groups) in a universe EF is of the form: ${µ}_{FG}:\mathrm{EF}\to \;\left[0,\;1\right]$, where ${µ}_{FG}\left(\mathrm{EF}\right)=s$ if s is the degree to which EF belongs to FG. The membership function takes values in the interval [0, 1]. Figure 5, for example, shows how values lower than 70% have a high degree of membership in the unacceptable fuzzy set and values greater than 100% have a high degree of membership in the fuzzy best-in-class set.

$$\mathrm{Membership Function}\;(x,\;[a,b,c,d])=\{\begin{array}{c} \begin{array}{c} 0,\;\;\;\;\;\;\;\;\;\;\;if\;x\leq a\;or\;x\;\geq d\\ ⃕\\ \frac{x-a}{b-a},\;\;\;\;\;\;\;if\;x>a\;and\;x<b\\ ⃕ \end{array}\\ 1,\;\;\;\;\;\;\;\;\;\;if\;b\;\leq \;\;\;\;\;x\;\;\;\;\;\;\;\leq c\\ \begin{array}{c} ⃕\\ \frac{d-x}{d-c},\;\;\;\;\;\;if\;x>c\;and\;x<d \end{array} \end{array}$$

Then, to forecast the VOP performance, the value effectiveness values are taken, to make a fuzzy qualification of them into five fuzzy groups.

In line with the above, a fuzzy rule (IF-Then) is expressed symbolically as:

IF < input variable is A > Then < output variable is B >.

Where < A and B > are descriptors of pieces of knowledge and rule articulates a relation between inputs and outputs, it is also possible to define a simple proposal of this type through:

p: IF X is A THEN Y is B.

**Figure 6:**
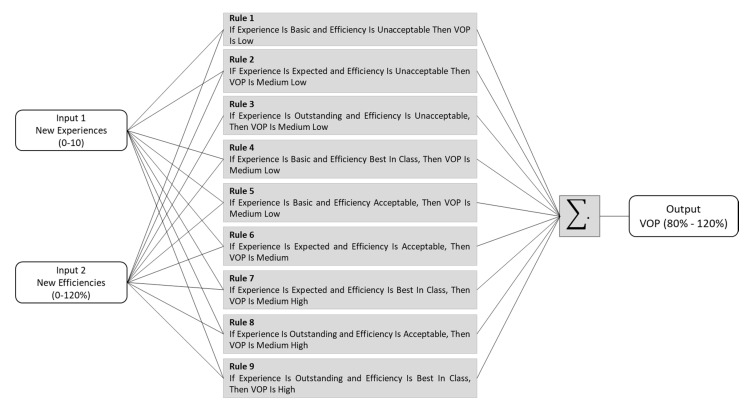
Rules of Fuzzy model.

The antecedent and the consequent of a rule could have multiple parts [44,47], in this way, fuzzy rules were divided to specify the appropriate logic mapping based on the value offer performance (VOP), the rules developed for this scenario are shown in Figure 6.

**Figure 7:**
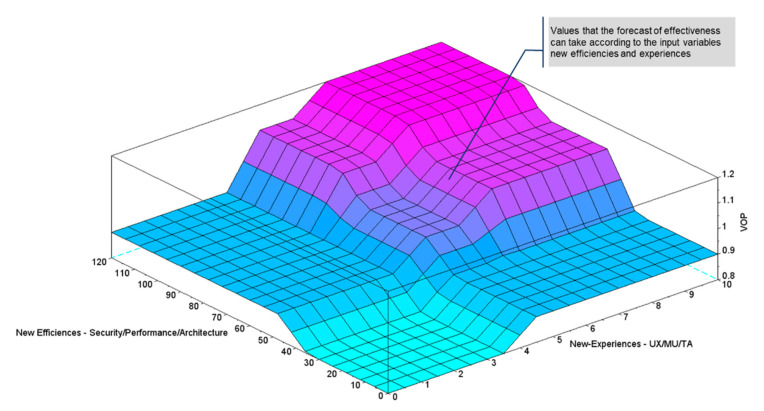
Fuzzy set.

After shaping the fuzzy group, the probability group could be analysed for the value offer to be Effective, in accordance with the new experiences (User experiences, actionable intelligence, and transitory advantage) and the new efficiencies of the platform (security, performance, and architecture). By creating a program in the Scilab software with the previously established parameters, the output surface can then be plotted, by means of the Plotsurf function, which has been executed with the following Plotsurf parameters (fis, [1 2], 1, [0 0]), in this way, the effectiveness behaviour in the defined fuzzy group can be observed in Figure 7.

#### 3.1.5. Input Variables

Two variable types enter this model, the first type are linguistic variables; they represent the new experiences of a business model and take values between the basic and the outstanding from the universe of discourse. The second entry variable which are the new effectiveness in the operative model, which take values from the speech universe between unacceptable and best in class.

#### 3.1.6. Outputs Variables

The exit variable in this model is the probability that the new experiences and effectiveness configurations generate value, that is, the perceptual value that the entry configuration scenario creates or destroys the value. That value scale and its impact on the value offer have been defined in the table above.

In summary, to model this subsystem, a composite index (TIN) was developed, which evaluates the technological management, in the same way, a fuzzy logic model was built to evaluate the performance of a digital value offer (VOP).

With the above in mind, the following sections explain the construction of a model based on system dynamics that seeks to identify the behaviour of value creation in an IoT ecosystem of the connected home.

### 3.2. Forrester Diagram

**Figure 8:**
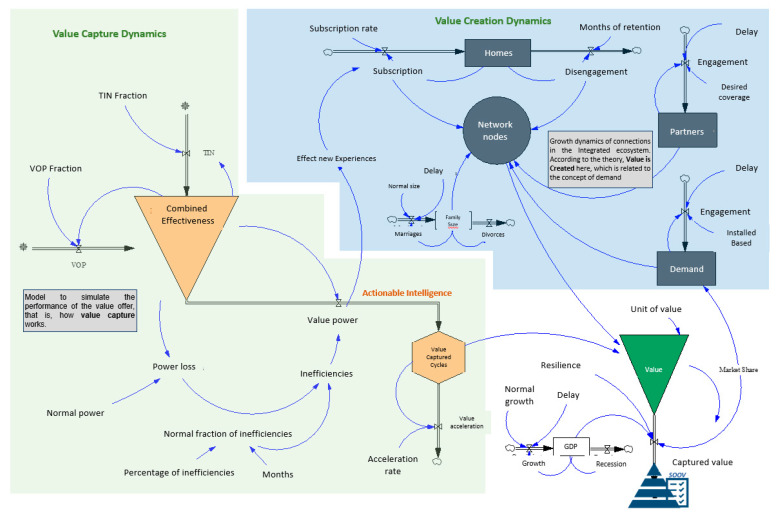
Forrester Diagram.

After conceptualizing the value creation system (see Figure 4) and with the evaluation subsystem defined in the previous section, the next objective was to develop a model that allowed to simulate the behaviour of the value. Subsequently, the Forrester diagram shown in Figure 8 was designed, which allowed for the writing of the equations in the computer in order to validate the model, observe the temporal evolution of the variables and perform sensitivity analysis. In 2020, due to the impact of the pandemic on the industry, different trends related to the value creation system have emerged; in fact, to face the pandemic and maintain sustainable businesses over time, there are some digital imperatives that do not wait, such as the search for new ways of operating leveraged on smart digitization [43].

Thus, in the digital value offer and technology management, the focus is on effectiveness, where the right synchronization of the concepts should allow for the creation of new experiences for the client and consumer. Furthermore, considering that the technological platform belongs to the integrated ecosystem, its management should also be effective and efficient.

Wherefore, for the Dynamic model development, the following considerations have been established:

1. The value creation dynamics are influenced by the number of new connections given in the ecosystem.
2. The technological platform is the integrated ecosystem enabler. There are two platforms that work together, one is oriented to the internal processes of the company and the other to the consumer (the connected home).
3. The configuration of the value offer and technology management defines the power of the value creation system.
4. The power (or theoretical performance) of the value creation system is moved in a fuzzy group of possible values.

The Forrester diagram (Figure 8), is also known as the flow diagram, which allows the model to be implemented in an IT tool, in this case, the Vensim software has been used.

The model was developed considering following: the three-part conceptual diagram mentioned previously; the value capture subsystem, where this process’ dynamics are simulated; and the value creation subsystem. Then, the value forecast subsystem is modelled using the results of these subsystems.

**Figure 9:**
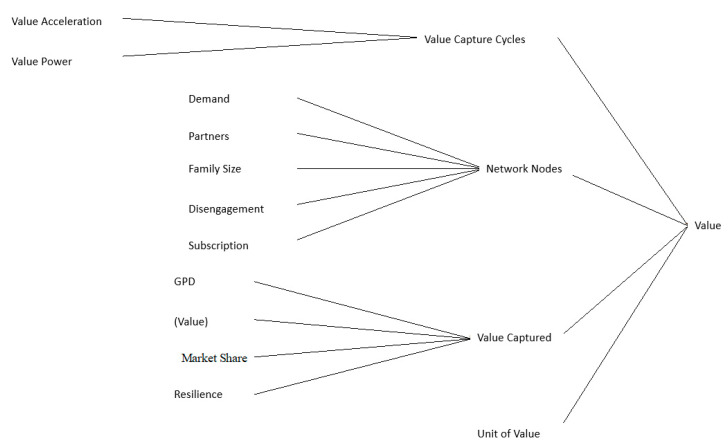
Causal Diagram.

Each of the subsystems has its respective feedback cycle, levels, and interrelations, which were used to execute the simulation and were refined with the sensibility and validation analysis of the dynamic hypothesis. The causal diagram of the model can be seen in Figure 9.

Vensim software has a function to check the model syntax (Check Model), through this functionality, the equation editor verifies that it uses all inputs, also checking that structure of the model is the same as that represented in the diagram. Next, to detect coherence errors in the units, the Units Check functionality was executed, in this way, it is guaranteed that in the iterative process of building, a verification of the model is carried out in parallel.

## 4. Discussion

The following sections explain the obtained results in the hundreds of simulations that were performed, these results were interpreted on the basis of an extended background review, with the observations made in the context of the white goods companies and with those provided by the experts. [48].

### 4.1. Simulation and Sensitivity Analysis

After verifying the parameters and their conceptual and numerical correspondence, the TIN (Technological Innovation Index) and VOP (Value Offer Performance) parameters were modified to represent values between −20% and 20% each (or what is the same between 80% and 120%).

**Figure 10:**
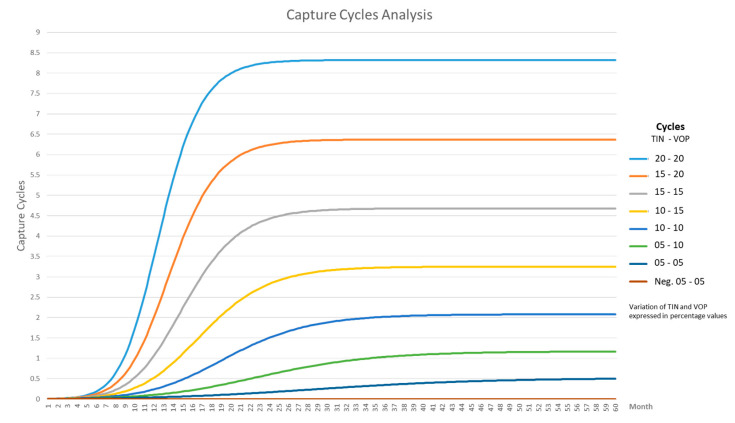
Capture cycle behaviour.

The subsystem’s value capture dynamics display behaviour that is increasingly logistic, which is represented in the increase of a variable when its density creates pressure over the intrinsic growth rate [49]. This behaviour is evidenced in the performed simulations and a summary can be observed in Figure 10.

**Figure 11:**
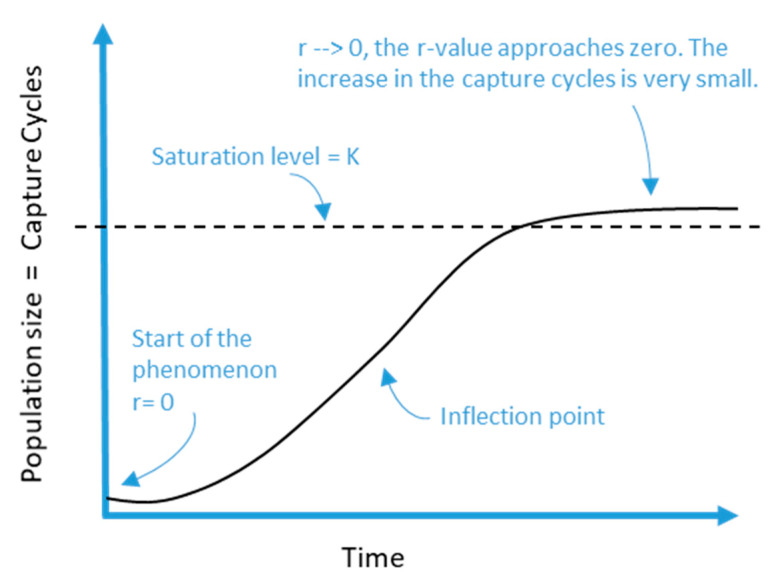
Graphic analysis of capture cycles.

Reflecting on Figure 10, it is possible to discover its relationship with the population growth curves of the logistic type, which represents the growth of a population when its density exerts some pressure on the intrinsic rate growth, as shown in Figure 11.

It was Pierre François Verhulst in 1838 who defined the equation to express this type of self-limited growth [49] and this can be seen below:

(1) $$R_{\left(t\right)}=\frac{K}{1+e^{c-rt}}$$

where

 *K* is the “loading capacity” of the system.
 *C* is the acquired constant in the integration process.
 *R*(*t*) is the number of capture cycles in the t time.
 *r* is the coefficient that indicates the magnitude of the growth potential of each value capture.

Table 2 has been generated by varying the effectiveness input parameter (combination of VOP and TIN), which was increased by one percentage unit at a time, generating a value of growth rate r and of maximum capacity K in each iteration. According to the results of the simulations and the adjustment of the model parameters, the limits were identified in Table 2 where the subsystem behaves stably; that is, it generates a positive degree in the value capture cycles.

In this subsystem, from the 5% of the effectiveness of the value offer, the capture cycles grow until they reach their limit 40% of effectiveness. In this way, the following values of the variables are identified for differential logistic Equation (1):

C=7.32

$$r_{n}=Potential\;rate\;\left(TP\right)=0.22\;\leq \;\;TP_{n}\;\leq \;0.57=\overset{0.56}{\underset{n=0.21}{\sum }}n+0.01$$

$$\begin{array}{ll} k_{n} & =0.1\;\leq \;k_{n\;}\leq \;\;8.32\\ & =\;-1.7107\;+\;0.2348\;*\;n\;+\;\left(n-18.5\right)\;*\;\left(\left(n-18.5\right)\;*\;0.0051\right) \end{array}$$

n=effectiveness of the value offer= 5 ≤ n ≤ 40

Replacing *k*, *C* and *r* in (1)

$$R_{\left(t\right)}=\frac{-1.7107\;+\;0.2348*\;n\;+\;(n-18.5)*((n-18.5)*0.0051)}{1+e^{7.32-\left({\mathrm{TP}}_{n}t\right)}}$$

$$R_{\left(t\right)}=\frac{-1.7107\;+\;0.2348*\;n\;+\;(n-18.5)*((n-18.5)*0.0051)}{1+e^{7.32-\left(\left(\sum_{n=0.21}^{0.56}n+0.01\right)t\right)}}$$

The solution of the logistic differential equation given above allows to identify the number of the system’s capture cycles. In other words, this equation predicts the possible value of R, given an effectiveness of the value offer and a period of time in the future.

In the context of connected homes, the previous results have special relevance in planning a company’s digital initiatives; understanding that the behaviour of the economic value is logistical and not exponential helps improve business cases before launching an initiative. Similarly, solving the mathematical equation of the curve allows to forecast the number of value capture cycles. This would enable companies to assess whether a digital initiative will be successful. The S-shaped logistics curve shows how the creation of value is affected by factors that limit unlimited growth; it can be deduced that this curve represents a behaviour more adjusted to the reality of digital businesses, especially of those operating in the field of connected homes.

### 4.2. Network Effect

The network effect is a phenomenon in the digital business model, which has been widely studied by different researchers. Iansiti and Lakhani [5] define it as “ the added value by the increase of the number of connections inside and trough the networks”, while others define it as the impact that the number of users of a platform has over the value created by each user and thus they are viewed as the main sources of value creation [50,51]. Tiwana [52] defines it as “the degree by which each platform or app user, makes it more valuable than the other existing users”. Economists denominate this effect as Network externalities or Metcalfe law.

In line with the above, the research of Zhang, Li [28] regarding the Chinese social network WeChat, shows that the indirect externalities, which are referred to as the accumulated benefit of the growing number of the network participants, affect the value of information significantly, but not the social value. That is to say, the accumulated benefit of the growing number of participants leads to the reduction of the average price and also impacts the amount of services that WeChat can offer [53]. On the contrary, when the auxiliary services are only used or consumed, they do not give social value, this implies that WeChat did not develop a competitive advantage over game suppliers, news, or stories.

In contrast, it is important to highlight that the size of the network determines the potential contacts with which a user can interact but does not guarantee the virtual connections that a user can build, nor does the utility provided of those connections. In this line, white goods companies encounter barriers in the implementation of large-scale systems, blocking the agility of the development of new digital-based offers. In information systems studies this is referred to as the resiliency that some internal users have against projects that imply new technologies [54,55]. Consequently, the search for new ways of interacting with the consumers should be one of the premises of value offers [56,57].

Analogously, it is important to mention that some of the investigators have been concerned about explaining the network effect and its impact on the competences. It is true that in praxis, some technological platforms have created monopolies that are hard to manage, so it can be inferred that public policies have not yet achieved the objective of avoiding these monopolies. This topic has become of high interest to the society in 2020 [58,59].

**Figure 12:**
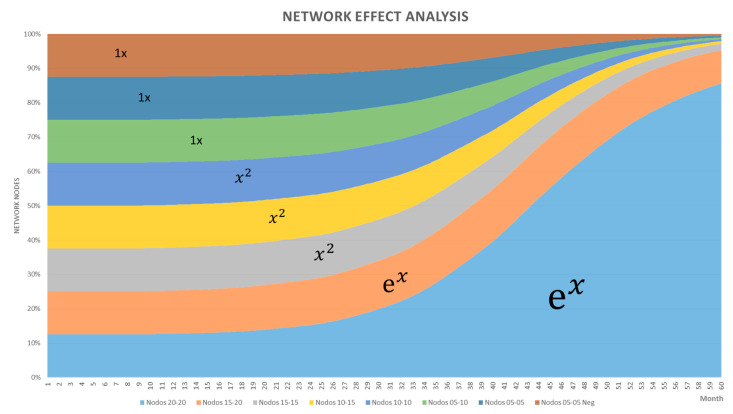
Network effect behaviour.

Concerning the dynamic simulation of the network effect in the modelled system, the detailed analysis of the variables shows that when the level of effectiveness is over 20%, approximately 80% of the network effect is generated, in one of the stable conditions of the system. Likewise, in the inferior effectiveness ranges, the network effect is practically linear with very low values, see Figure 12. It is logical then to think that when the value offer has an acceptable performance, the network effect grows linearly, as the value offer grows in effectiveness it can pass to a network effect whose curve has a quadratic shape, however with outstanding effectiveness can be achieved exponential network effects, something that will surely excite the business leaders of the companies.

#### Demand Estimation in White Goods Companies

Several theories and models exist regarding the execution of the demand estimation process, from the application of basic rules for data organization and prediction based on ratios, to the application of neuroeconomic techniques. Nonetheless, within the academic community there are for and against arguments regarding the use of some techniques. There is a consensus regarding the time series regression algorithms, which generate one of the best-forecasted results. Likewise, neural networks can be used in praxis and several scientific papers have been written regarding their application in this area [60].

In line with the above, it is important to mention that the results provided by forecast model depend, to a large degree, on the data. A structured method is required to organize and process the data. In this way, it is possible to get knowledge of the data and their characteristics, making it possible to extract information that will have a positive impact on decisions making.

According to the literature review, the demand for appliances is closely related to the economic performance of a country [61]. In this regard, several studies confirm that there is also a correlation between the home sells, marriages and divorces, and the appliances replacement, which represents almost the total of the demand for these products [62]. Thus, the new practical review confirms that there are multiple regression algorithms that are the most used ones in predicting the demand for refrigerators. When combining their results with artificial intelligence methods, the increase in assertiveness does not exceed 5% [63].

Taking into account the above, it can be confirmed then that the ARIMA algorithms are the ones that represent better results regarding the refrigerator demand forecast. This type of models has a combination of auto-regressive and mobile media, that search for time series patterns and make predictions. In general terms, the function that represents these algorithms is described as follows:

(2) $$y_{t}=µ+\varphi_{1}y_{t-1}+\ldots +\varphi_{p}y_{t-p}+\theta_{1}e_{t-1}+\ldots +\theta_{q\;}e_{t-q}+\;e_{t}$$

where

 µ Is a constant.
 $\varphi_{1}y_{t-1}+\ldots +\varphi_{p}y_{t-p}$ Are the auto-regressive terms (lagged values of y).
 $\theta_{1}e_{t-1}\ldots -\theta_{q\;}e_{t-q}$ Are the mobile media terms (lagged mistakes).
 $e_{t}$ Error or stochastic disturbance.

In conclusion, it can be affirmed then that in spite of the existing limitations among the different forecast models, the network effect can be reduced to a demand forecast for the products that a white goods company sells and, in this way, the model is broadened.

### 4.3. Value Forecast Analysis

Finally, it can be inferred that the relations between the subsystems define the theoretical quantity of a project forecasted to the future, in this sense, the used formula in this model is the following:

*Value(t)* = *((Value Capture Cycles × Network Effect × Value Unit) − Captured value)* (3)

The captured value is represented as a total value fraction that the system could generate, this must be limited based on the market share of the company. In other words, the capture capacity is influenced by the market share of the company. Hence, it is possible to infer that the bigger market share, the bigger the value capture capacity. As mentioned before, the economic performance of a white goods company is directly related to the economic performance of a country [62], thus, the Gross Domestic Product behaviour is integrated in the model. Likewise, the resiliency capability of the system could generate a delay in the value capture, so it was necessary to integrate it into the simulation model.

**Figure 13:**
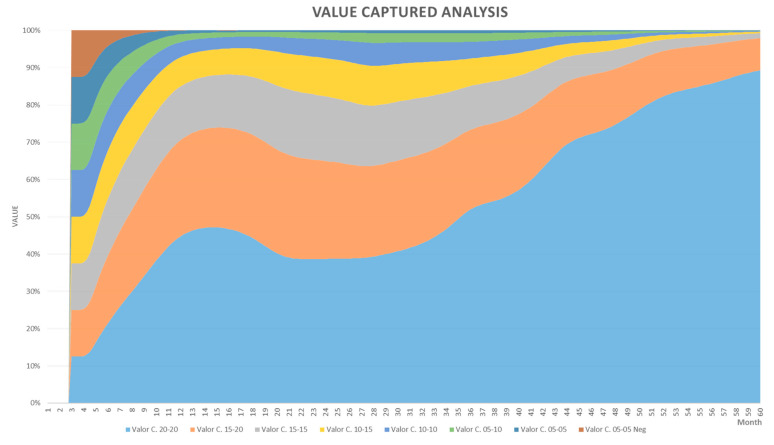
Growth of captured value.

The simulation results show a quadratic type value growth curve, however, in an environment where GDP goes around 5%, and the resiliency reflects a capture delay of 2 months, the curve represents the fluctuations that are shown in Figure 13.

In the same way, the behaviour reflected in the network node growth is also shown in the quantity value, where effectiveness rates higher than 20% increase the probability to capture more value. Nevertheless, the model does not behave in an exponential way.

In this way, the t month value is defined as the theoretical value that each home generates multiplied by the network effect of the new homes connected to the IoT system. White goods companies have made apparently logical research on value based on the return of investment calculation (ROI) (Although it is important to clarify that this perspective is one of the most paralyzing for the adoption of IoT by companies). Nevertheless, there is a large amount of reasons for which it is very difficult to obtain correct estimations during the first days of any new technology [64]. In this regard, approach to the economic value created for each home is based on the premise that in a manufacturing company, the projects involving investment in new business use the financial concept of the balance point, to know prior to starting the operations the level of income required to recover the investment must be known, thus, the value per home is defined as follows:

(4) $$Value\;per\;home=\frac{Fixed\;cost\;per\;home}{\mathrm{Contribution}\;\mathrm{margin}\;\mathrm{per}\;\mathrm{home}}$$

where

- *Value per home* = income obtained through the commercialization of digital services in the home.
- *Fixed cost per home* = are the expenses that remain constant despite the quantity of commercialized services.
- Contribution margin per home = the difference between the incomes (billing) and other variable costs.

Thus, considering that the balance point is given by a *Value per Home* = 1, value projection is inferred, so that:

*Value per home* = *Value unit*

where *Value unit* (VU) = Theoretical Value that generates the information flow (actionable intelligence flow) in the system, it is important to highlight that, the *actionable intelligence flow* is the main component of the business model scenario to be tested; for example, a preventive maintenance Service for appliances, a cooking recipe, automatic grocery ordering, auto replenishment of appliances in a retail chain (retail), etc. [65].

Simplifying the Equation (3):

$$Value_{t}=\left(VU*\;R*N\right)-VC$$

where

 $Value_{t}=$ Value quantity forecast.
 *VU* = Value unit, represent the theoretical value of an actionable intelligence Flow.
 *R* = Value capture cycles.
 *N* = Network effect calculation.
 VC = Captured value.

## 5. Theoretical Implications

The theoretical foundations of the business model based on data are crystalized in a value offer to the client. The characteristic of this value offer is its digital component and a mediated delivery through a technological platform, this one being the company’s property or a third party technological platform [8,66,67,68]. This value offer stimulates the creation of the ecosystem’s value. The value proposition is the guide by which the company organizes itself to execute the different operations that have to be done to meet the customer’s needs [69,70]; that is, the mechanisms by which the value creation system is stimulated so that its goal can be accomplished.

The results obtained in this investigation confirm hypothesis on the dynamic value offer effect in value creation and presents new elements for improved understanding of this complex system. It can be concluded that the traditional valuation laws are based on certain assumptions that not necessarily apply to the context of Connected Homes, so one of the contributions of this investigation is that it has proposed an adapted conceptualization for this ecosystem. Hence, the existence of diverse reference frameworks for the design of digital business cases has been evidenced; thus, to complete these frameworks with The results obtained in this investigation will strengthen those frameworks and make them more complete, making it possible to develop an indicator that monitors long-term behaviour.

## 6. Conclusions

In a world where business uncertainty has increased due to Covid-19, there is no right answer as to how the collision between manufacturing and digital business companies is to be faced. Despite this, we must keep searching for solutions; the results of this research provide scientific mechanisms for analysis and design, enabling resilient operating models. The integration of the IoT technology platform that supports the operating model, as well as the value offer, has led to the creation of a holistic and comprehensive vision, which is materialized in the conceptualization of the system and in the proposed simulation model. This model has made it possible to identify the behaviors of the value creation system at the beginning of this research, which would not have been possible otherwise.

From the methodological perspective, the application of systems theory in the analysis of the object of the study, has made it possible to conceptualize a value creation model for the IoT ecosystem. Similarly, the ideas, relationships and concepts identified in the theory helped refine the model and successfully integrate the proposed subsystems. The expert study and the critical analysis of documents have led to the inclusion of new elements in the construction of system representation, moving on from the conceptualization-generalization to the modelling through the system dynamics.

Furthermore, the Forrester diagram is an excellent tool for the analysis of the causality present in the value creation system, so it has been possible to validate the model and to observe the temporary evolution of the value creation and capture subsystems. Thanks to the results it has been possible to mathematically formulate the behaviour and obtain a general formula that allows to estimate the quantity of value that the system generates in a period of t time.

Likewise, the sensibility analysis allowed us to discover the behaviour of the value creation and capture subsystems, the former is influenced by the network effect, which in turn is related to demand-related behaviour. In this regard, the curve that represents this phenomenon shows that at small effectiveness values the growth is practically linear, and at intermediate levels, it behaves in a quadratic way. However, at outstanding levels, the curve represents exponential behaviour. In the case of the value capture subsystem, it has been discovered that this component is responsible for potentiating or diminishing the value capture capability, so at outstanding effectiveness levels, the logistic curve reaches its maximum capability, ultimately impacting the value quantity that could be captured for a specific offer under established circumstances.

From a practical point of view, understanding value creation- and capture-related behaviors in the IoT ecosystem of Connected Homes is a big step forward. It will allow white goods companies to adapt their operative models and to design a digital-based value offer, which could include the concepts described here from the beginning and hence would have more probability of capturing economic value.

Moreover, the union of the production and consumption ecosystems allows to integrate value chain objectives in these digital initiatives, something that is not a common praxis. This would enable the companies to adapt their business models beyond appliance sale and after-sale service. Additionally, this paper contributes to the development of the digital capacities of a company and to our understanding of how created value is a differentiating factor that leverages the development of competitive advantage for any enterprise.

The new normal and efficiency creation in the ecosystem are some of the challenges that white goods companies have to face in 2020. Regarding this challenge, the fuzzy logic leveraged the development of a model that prevents uncertainty associated with value offer evaluation. Its practical application allowed to line the value creation system entry concepts and worked as a basis for the development of computer applications that support the digital transformation of white goods companies.

In practice, an enterprise’s understanding of the integrated IoT ecosystems’ dynamics allows it to include transient advantage in its strategy, designing offers in accordance to the value creation and capture dynamics. The results of this investigation show that to reach the maximum value offer potential, it is necessary to reach or to exceed the evaluation of the offer, which will drive the value captured and therefore the results of the company will be as good as possible.

This investigation is part of a bigger one that includes the IoT ecosystem design in the Connected Home context, the results obtained from the modelling and simulation, support the structuring of a new methodology that allows white goods companies to study scenarios through value offer validation.

## Figures and Tables

**Table 1:** Value Offer Performance description.

VOP	Fuzzy Groups—FG	Impact on Value Creation
VOP < 80%	Unacceptable	Value creation is not sufficient to sustain a balance point. Unacceptable offer competitiveness.
80% < VOP < 90%	Low	Value creation is low, the offer design mistakes should be corrected.
90% < VOP < 100%	Acceptable	Value creation has some slight variations, tending toward low, which leads to the revaluation of the competitiveness offer
100% < VOP < 110%	Outstanding	An acceptable competitive advantage has been achieved; the value creation engine is in the outstanding performance range.
VOP > 110%	Best in Class	A transient advantage has been achieved, the value creation in the system is excellent, the value development engine is at its highest potential.

**Table 2:** Limits of the System.

Effectiveness = VOP and TIN Combination	Growth Rate = r	Maximum Capacity = K	Effectiveness = VOP and TIN Combination	Growth Rate = r	Maximum Capacity = K	Effectiveness = VOP and TIN Combination	Growth Rate = r	Maximum Capacity = K
5%	0.22	0.10	17%	0.34	1.50	29%	0.46	4.37
6%	0.23	0.15	18%	0.35	1.68	30%	0.47	4.68
7%	0.24	0.22	19%	0.36	1.88	31%	0.48	5.00
8%	0.25	0.30	20%	0.37	2.08	32%	0.49	5.32
9%	0.26	0.40	21%	0.38	2.29	33%	0.50	5.66
10%	0.27	0.50	22%	0.39	2.52	34%	0.51	6.01
11%	0.28	0.61	23%	0.40	2.75	35%	0.52	6.37
12%	0.29	0.74	24%	0.41	3.00	36%	0.53	6.74
13%	0.30	0.87	25%	0.42	3.25	37%	0.54	7.12
14%	0.31	1.01	26%	0.43	3.52	38%	0.55	7.51
15%	0.32	1.17	27%	0.44	3.79	39%	0.56	7.91
16%	0.33	1.33	28%	0.45	4.08	40%	0.57	8.32

## Data Availability

Data available on request due to restrictions e.g., privacy or ethical. The data presented in this study are available on request from the corresponding author.
